# Overestimation of the Apparent Diffusion Coefficient in Diffusion-Weighted Imaging Due to Residual Fat Signal and Out-of-Phase Conditions

**DOI:** 10.3390/tomography12010011

**Published:** 2026-01-16

**Authors:** Maher Dhanani, Dominika Skwierawska, Tristan Anselm Kuder, Sabine Ohlmeyer, Michael Uder, Sebastian Bickelhaupt, Frederik Bernd Laun

**Affiliations:** 1 Institute of Radiology, Uniklinikum Erlangen, Friedrich-Alexander-Universität Erlangen-Nürnberg, 91052 Erlangen, Germany; maherdhanani@gmail.com (M.D.); dominika.skwierawska@uk-erlangen.de (D.S.); sabine.ohlmeyer@uk-erlangen.de (S.O.); michael.uder@uk-erlangen.de (M.U.); sebastian.bickelhaupt@uk-erlangen.de (S.B.); 2 Department of Medical Physics in Radiology, German Cancer Research Center, 69120 Heidelberg, Germany; t.kuder@dkfz.de

**Keywords:** diffusion MRI, fat suppression, *ADC* overestimation

## Abstract

**Background/Objectives**: Diffusion-weighted imaging (DWI) is a magnetic resonance technique used to map the apparent diffusion coefficient (ADC) of water in human tissue. ADC assessment plays a central role in clinical diagnostics, as malignant tissues typically exhibit reduced water mobility and, thus, lower ADC values. Accurately measuring the ADC requires effective fat suppression to prevent contamination from the residual fat signal, which is commonly believed to cause ADC underestimation. This study aimed to demonstrate that ADC overestimation may occur as well. **Methods**: Our theoretical analysis shows that out-of-phase conditions between fat and water signals lead to ADC overestimations. We performed demonstration experiments on fat–water phantoms and the breasts of 10 healthy female volunteers. In particular, we considered three out-of-phase conditions: First and second, short-time inversion recovery (STIR) fat suppression with incorrect inversion time and incorrect flip angle, respectively. Third, phase differences due to spectral fat saturation. The ADC values were assessed in regions of interest (ROIs) that included both water and residual fat signals. **Results**: In the phantoms and the volunteer data, ROIs containing both fat and water signals consistently exhibited lower ADC values under in-phase conditions and higher ADC values under out-of-phase conditions. **Conclusions**: We demonstrated that out-of-phase conditions can result in ADC overestimation in the presence of residual fat signals, potentially resulting in false-negative classifications where malignant lesions are misinterpreted as benign due to an elevated ADC. Out-of-phase fat and water signals might also reduce lesion conspicuity in high b-value images, potentially masking clinically relevant findings.

## Abstract (plain language summary)

Diffusion-weighted MRI helps doctors distinguish benign from malignant tissue by measuring how easily water moves within tissue. Cancer usually restricts water movement, leading to lower measured water mobility values. This study shows that leftover fat signal can sometimes falsely increase the measured water mobility. Using experiments in test objects and healthy volunteers, we provide evidence that this effect may make cancers appear harmless or harder to see. Recognizing this source of error may improve MRI accuracy and guide better imaging methods in clinical practice.

## 1. Introduction

Diffusion-weighted magnetic resonance imaging (DWI) has become a cornerstone of modern radiology [1,2,3,4,5,6,7,8,9,10,11]. It can be used to measure the apparent diffusion coefficient (ADC) of water, which reflects the mobility of water molecules as they undergo diffusive motion within human tissue. The ADC is particularly relevant because it is sensitive to diffusion barriers, such as cell membranes. Consequently, malignant tissues, which often exhibit increased cellular density and more restricted extracellular space, typically exhibit lower ADC values. This characteristic reduction in the ADC is widely used in clinical diagnostics to help identify and assess suspicious lesions [12,13,14,15,16,17]. Therefore, in most clinical contexts, a *decrease* in the water ADC serves as a key indicator when evaluating tissue abnormalities.

The presence of fat is highly relevant for image interpretation. Because fat molecules are significantly larger than water molecules, the ADC of fat is approximately two orders of magnitude lower than the ADC of water [18,19]. Consequently, a low measured ADC value in an image does not necessarily indicate restricted diffusion due to pathology—it can also result from signal contributions from fat.

To mitigate the fat contamination issue, the use of fat suppression techniques is essential in DWI [1,13,20]. Without fat suppression, the measured ADC reflects a fat–water averaged value rather than the true water-specific ADC. This spurious ADC measurement provides far less information about the underlying tissue structure and type and is strongly influenced by the relative proportions (partial volumes) of fat and water within the voxel.

If the fat suppression is incomplete, the residual fat signal may remain in the diffusion-weighted images. This residual fat signal is commonly believed to result in an *underestimation* of the ADC [13,21,22,23,24,25,26].

The notion that the contaminating fat signal lowers the measured water ADC is entirely reasonable since the ADC obtained from an image voxel represents a signal-weighted average of the ADC values of all tissues or subcompartments present within that voxel [27]. However, there is one subtility to this notion. In magnetic resonance imaging (MRI), the signal is generally complex. For example, this is exploited in Dixon MRI techniques that can separate fat and water signals based on their different Larmor frequencies [28]. From Dixon MRI, it is also well known that the water and fat signals can cancel each other out in so-called out-of-phase images.

Similar out-of-phase conditions can occur due to a variety of reasons in DWI.

For example, they can occur with short-time inversion recovery (STIR) fat suppression [29]. As exploited in phase-sensitive inversion recovery experiments [30,31,32,33], the phase of the magnetization can be altered with inversion recovery preparations. Here, it is not the difference in resonance frequencies that is relevant, but the different longitudinal relaxation behavior. In an STIR preparation, the magnetization of fat is initially inverted using an inversion pulse, flipping it so that it points opposite the main magnetic field [29,34], making it negative in that sense. Then, the magnetization increases again due to T_1_ relaxation until it is aligned with the magnetic field, becoming positive. In a perfect scenario, the water magnetization is excited exactly at the time point when the fat magnetization becomes zero on its path from negativity to positivity, so that fat does not contribute to the signal. In practice, however, it is rarely possible to hit this zero-crossing time point with perfect accuracy.

A second potential issue is that B_1_^+^ field inhomogeneities and miscalibrations could lead to imperfect flip angles in certain voxels, causing the fat magnetization to be positive at the inversion time, while water magnetization remains negative due to its longer T_1_ time. This can also result in an opposed-phase condition, similar to that seen in out-of-phase Dixon imaging, where fat and water signals partially cancel each other out.

A third source of out-of-phase conditions between fat and water signal may be the use of effectively different pulse phases of spectral fat suppression pulses and water excitation pulses.

While many previous studies have focused on in-phase conditions, in this study, we demonstrate that such out-of-phase conditions may result in an *overestimation* of the ADC for the three mentioned cases.

## 2. Materials and Methods

### 2.1. Theory

#### 2.1.1. Influence of Out-of-Phase Conditions on the Measured ADC in Fat/Water Partial Volume Situations

The signal $S\left(b\right)$, as a function of the diffusion encoding b, can be approximated with a two-compartment model in a fat/water partial volume situation:

(1) $$S\left(b\right)=S_{tissue}\exp\left(-b\cdot D_{tissue}\right)+S_{fat}\exp\left(-b\cdot D_{fat}\right).$$

Here $D_{tissue}$ and $D_{fat}$ are the ADC values of tissue and fat, respectively, and $S_{tissue}$ and $S_{fat}$ are the signals originating from tissue and fat, respectively. The ADC is computed as:

(2) $$D_{app}=-\frac{1}{b}\log\frac{S\left(b\right)}{S\left(0\right)}.$$

Equation (1) results in the following two-compartment ADC (assuming $D_{fat}\approx 0$) if the diffusion encoding is sufficiently small so that $\exp\left(-b\cdot D_{tissue}\right)\approx 1-b\cdot D_{tissue}$:

(3) $$\begin{array}{ll} D_{app}=-\frac{1}{b}\cdot \log & \frac{S_{tissue}\exp\left(-b\cdot D_{tissue}\right)+S_{fat}\exp\left(-b\cdot D_{fat}\right)}{S_{tissue}+S_{fat}}\\ & \approx -\frac{1}{b}\cdot \log\frac{S_{tissue}\cdot \left(1-b\cdot D_{tissue}\right)+S_{fat}}{S_{tissue}+S_{fat}}\\ & =-\frac{1}{b}\cdot \log\left(\frac{S_{tissue}+S_{fat}}{S_{tissue}+S_{fat}}-b\frac{S_{tissue}D_{tissue}}{S_{tissue}+S_{fat}}\right)\\ & =-\frac{1}{b}\cdot \log\left(1-bD_{partial\;volume}\right)\\ & \approx -\frac{1}{b}\cdot \log\left(\exp\left(-b\cdot D_{partial\;volume}\right)\right)\approx D_{partial\;volume}. \end{array}$$

If the water and the fat signal are in phase, then $S_{tissue}$ and $S_{fat}$ have the same algebraic sign and, consistent with the references [13,21,22,23,24,25,26], the measured partial volume affected apparent diffusion coefficient

(4) $$D_{partial\;volume}≔\frac{S_{tissue}}{S_{tissue}+S_{fat}}\cdot D_{tissue}=\frac{\left|S_{tissue}\right|}{\left|S_{tissue}\right|+\left|S_{fat}\right|}\cdot D_{tissue}$$

is smaller than $D_{tissue}$.

If the water and the fat signal are out-of-phase, then $S_{tissue}$ and $S_{fat}$ have opposite algebraic signs and one finds

(5) $$D_{partial\;volume}=\frac{S_{tissue}}{S_{tissue}+S_{fat}}\cdot D_{tissue}=\frac{\left|S_{tissue}\right|}{\left|S_{tissue}\right|-\left|S_{fat}\right|}\cdot D_{tissue}\approx \left(1-\frac{\left|S_{fat}\right|}{\left|S_{tissue}\right|}\right)\cdot D_{tissue}>D_{tissue}.$$

In the last step of this computation, it was assumed that the fat signal is smaller than the water signal so that the formula ${\left(1+x\right)}^{-1}\approx 1-x$ can be used.

Thus, in an out-of-phase condition, $D_{partial\;volume}$ can be larger than $D_{tissue}.$

#### 2.1.2. STIR with Incorrect Inversion Time

With STIR preparation, signals must be weighted by the factor $c=1-2\exp\left(-TI/T_{1}\right)$ to account for T_1_ relaxation effects, with the inversion time TI. For tissue, $c_{tissue}<0$, since $T_{1,tissue}>TI$, yielding $S_{tissue}<0$. Ideal fat suppression implies $c_{fat}=0\to S_{fat}=0$.

Case 1: If TI is too short, $c_{fat}<0\to S_{fat}<0$, and $S_{tissue}$ and $S_{fat}$ have the same algebraic sign. This is an in-phase condition.

Case 2: If TI is too long, $c_{fat}>0\to S_{fat}>0$, and $S_{tissue}$ and $S_{fat}$ have different algebraic signs. This is an out-of-phase condition.

#### 2.1.3. STIR with Incorrect Flip Angle

For general flip angles α, the *c*-factor becomes $c=1+\left(\cos\alpha -1\right)\cdot \exp\left(-TI/T_{1}\right).$ If TI is set to $\ln2\cdot T_{1,fat}$, the value that ensures fat suppression at α = 180° in the long repetition time limit, the *c*-factor becomes:

(6) $$c_{fat}=1+\left(\cos\alpha -1\right)\cdot \exp\left(-\frac{\ln2\cdot T_{1,fat}}{T_{1,fat}}\right)=1+\left(\cos\alpha -1\right)\cdot \exp\left(-\ln2\right)\;=1+\frac{\cos\alpha -1}{2}=\frac{1+\cos\alpha }{2}.$$

At α=180°, $c_{fat}=(1+\cos\alpha )/2$ becomes zero and a perfect fat suppression is achieved. Notably, $c_{fat}$ has a minimum at α=180° as can be seen from computing $c_{fat}$ for α=180°+Δα:

(7) $$c_{fat}=\frac{1+\cos\left(180{}^{\circ}+\Delta \alpha \right)}{2}=\frac{1-\cos\left(\Delta \alpha \right)}{2}.$$

Consequently, an incorrect flip angle leads to a positive $c_{fat}$-factor in both cases when α is too small or too large.

In contrast, for reasonably small flip angle errors, $c_{tissue}$ will always be negative. Thus, a flip angle error will always lead to an opposed phase condition between fat and water signals if the nominal flip angle is set to 180° in the sequence. Consequently, the ADC is overestimated for positive and negative Δα.

#### 2.1.4. Spectral Fat Saturation

If the fat excitation pulse in a spectral fat suppression module effectively has a different pulse phase than the actual water excitation pulse, an out-of-phase condition may arise as well. This would become visible, if the spoiler gradients do not achieve a complete suppression of the fat signal. For example, if the water excitation pulse has the phase zero and the fat excitation pulse effectively has the effective phase $\theta_{fat}$, then the respective c-factors are 1 and $\exp\left(i\theta_{fat}\right)$. Here, we used the word “effective” to account for the fact that the magnetization’s phase offset can arise from two effects: the actual pulse phases and the fat/water dephasing that occurs between the fat rf pulse and the water excitation pulse.

Moreover, if a miscalibration occurs and the spectral fat saturation RF pulse is applied with a flip angle larger than 90°, the longitudinal magnetization that remains after the spoiler gradients is inverted. Similarly to the discussed STIR cases, this also leads to an opposed phase condition between the fat and water signal.

#### 2.1.5. Signal at High b-Values

The opposed phase condition also affects the partial volume signal. In an opposed phase condition, where $S_{tissue}<0$ and $S_{fat}>0$, the signal is:

(8) $$S\left(b\right)=-\left|S_{tissue}\right|\exp\left(-b\cdot D_{tissue}\right)+\left|S_{fat}\right|\exp\left(-b\cdot D_{fat}\right).$$

A numerical example may help to demonstrate the consequences of this relationship: If $S_{tissue}=100$ and $S_{fat}=$ 10 with $D_{tissue}=$ 1 µm^2^/ms and $D_{fat}\approx$ 0 µm^2^/ms, then, at b = 1500 s/mm^2^,

- The absolute pure tissue signal is 100 exp(−1.5) ≈ 22;
- The absolute pure fat signal is approximately 10;
- The absolute partial volume signal is 32 in an in-phase condition, and 12 in an opposed phase condition.

Thus, a lesion can appear darker on a high-b-value image when an opposed-phase condition is present (with a signal of 12 instead of 22). Moreover, it may become invisible because it has approximately the same signal as the fat signal, which is 10 in this example. This problem does not arise in an in-phase condition (lesion signal of 32 instead of 22 versus fat signal of 10).

### 2.2. Phantom Exams

We performed demonstration experiments for the three considered cases.

**Figure 1:**
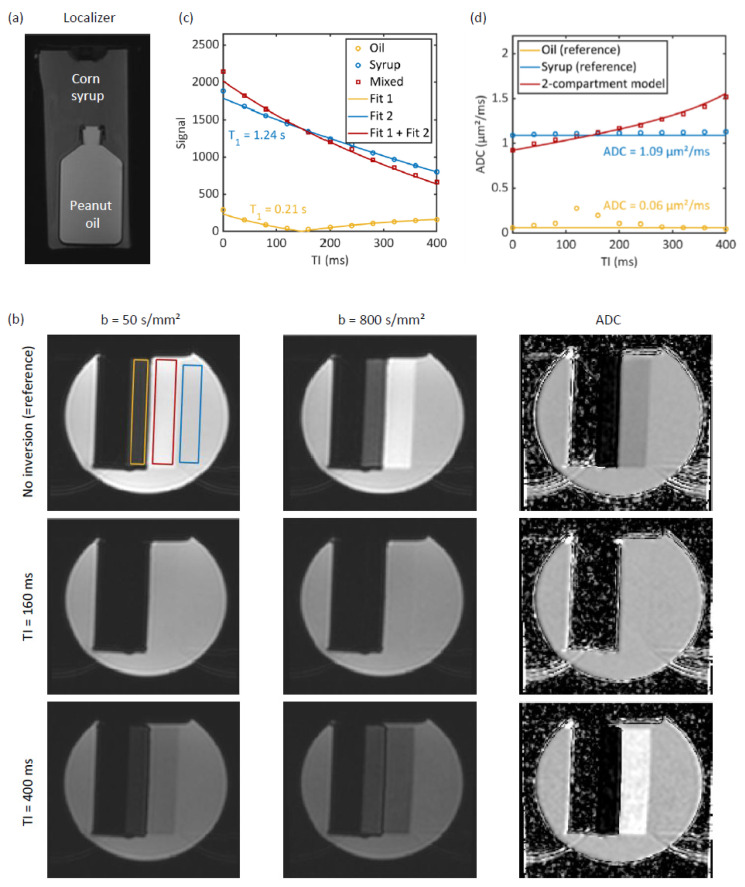
Phantom experiments. (**a**) Localizer image. The corn syrup solution mimics water in tissue, and peanut oil mimics fat tissue. (**b**) Images acquired at b = 50 and 800 s/mm^2^ and apparent diffusion coefficient (ADC) maps. Three regions of interest (ROIs) are depicted: pure oil (yellow), pure corn syrup solution (blue), and a mixed region (red) that arose due to the chemical shift. Without inversion recovery preparation (reference), the ADC is reduced in the mixed region, while it is increased for the TI = 400 ms case. (**c**) T_1_ fit with the ROI-averaged signals obtained from b = 50 s/mm^2^ images. The circular markers represent the measured values. (**d**) ADC values measured in the phantom. The red line represents the two-compartment model (Equation (1), parameters set to the previously obtained fit parameters: $T_{1,syrup}$ = 1.24 s, $T_{1,oil}$= 0.21 s, ${ADC}_{syrup}$ = 1.09 µm^2^/ms, ${ADC}_{oil}$ = 0.06 µm^2^/ms).

For the inversion time experiments, we measured a cylindrical phantom (“phantom 1”) filled with a 40% weight-by-weight water–corn syrup solution containing CuSO_4_ to prevent mold (Figure 1a). Corn syrup (ACH Food Companies, Inc., Cordova, TN, USA) was used to mimic water in tissue (as in Keenan et al. [35]) and peanut oil (vita Erdnussöl; Brändle, Empfingen, Germany) to mimic fat tissue (as in Hansmann et al. and Niebuhr et al. [24,36]). A peanut oil-filled bottle was immersed into the cylinder. The exams were performed at 1.5 T on a Magnetom Aera MRI scanner (Siemens Healthineers, Erlangen, Germany) with a body 18 coil and spine 72 coil. The acquisition parameters are summarized in Table 1. In short, we used a range of inversion times to demonstrate the effect of TI on the ADC and to measure the T_1_ times. The smallest b-value was set to 50 s/mm^2^ to minimize the influence of intravoxel incoherent motion effects in the volunteer exams.

For the flip angle experiments, we measured a smaller bottle filled with a 40% weight-by-weight water–corn syrup solution and peanut oil, which formed two separate phases with the oil floating on top of the syrup (“phantom 2”). In the flip angle experiments, a vial filled with peanut oil was placed next to phantom 2. These exams were performed at 3 T on a Magnetom Cima.X MRI scanner (Siemens Healthineers, Erlangen, Germany) with a 20-channel head coil. The acquisition settings are also summarized in Table 1. The acquisition settings were chosen such that peanut oil signal of the vial shifted into the corn syrup region of phantom 2. The flip angle of the inversion pulse was adapted by adapting its pulse voltage. The pulse voltage of the other RF pulses was kept fixed. In a real setting, where the transmit field varies over the field of view, this would be different: The flip angles of all pulses would scale in the same manner. Here, we nonetheless decided to isolate the inversion pulse flip angle dependency to demonstrate the effect more clearly. The nominal voltage indicated as by the scanner was 321 V. We used the voltages 90 V, 110 V, 150 V, 250 V, and 321 V.

For the spectral fat saturation experiments, we also used the Cima.X scanner and phantom 2, but without the peanut oil filled vial. Table 1 states the acquisition settings. We used one slice positioned at the interface of the two fluids. Images were acquired once with spectral fat suppression and once without (in this case, the voltage of the spectral fat suppression pulses was set to zero).

For the phantom IR experiments, the data were evaluated quantitatively in MATLAB (version 2022b; MathWorks, Natick, MA, USA).

The ADC values and signals were measured in rectangular regions of interest (ROIs, Figure 1b) in the corn syrup solution and oil, and in overlapping regions that arose due to the chemical shift. For this purpose, we used the ADC map generated by the scanner.

The T_1_ times of the corn syrup solution and oil were fitted using the respective ROI-averaged signals of the b = 50 s/mm^2^ images with the MATLAB function *fit* and the fit equation $abs(1-2exp\left(-TI/T_{1}\right))$ using the IR measurements. The fit also provided 95% confidence intervals (CIs).

Using these fitted T_1_ times, the theoretical ADC values were computed for all TIs with Equation (2),

(9) $$ADC=-\frac{1}{b}\log\frac{S\left(b=800\frac{s}{{mm}^{2}}\right)}{S\left(b=50\frac{s}{{mm}^{2}}\right)},$$

where the $S\left(b\right)$ expression in Equation (1) was used. For this purpose, $S_{tissue}$ and $S_{fat}$ of Equation (1) were set to the ROI-averaged signals of the corn syrup solution and oil, respectively. The ADC values that were obtained without STIR preparation were used in this computation.

### 2.3. Volunteer Exams (Incorrect Inversion Time)

We performed breast examinations on 10 healthy female volunteers aged 24–26 years in 2024, who provided written informed consent to participate in this prospective IRB-approved study (approval number 61-21 B). This sample size was chosen based on current standard practice [37]. The exams were performed at 3 T on a Magnetom Vida MRI scanner (Siemens Healthineers, Erlangen, Germany) with an 18-channel breast coil.

**Figure 2:**
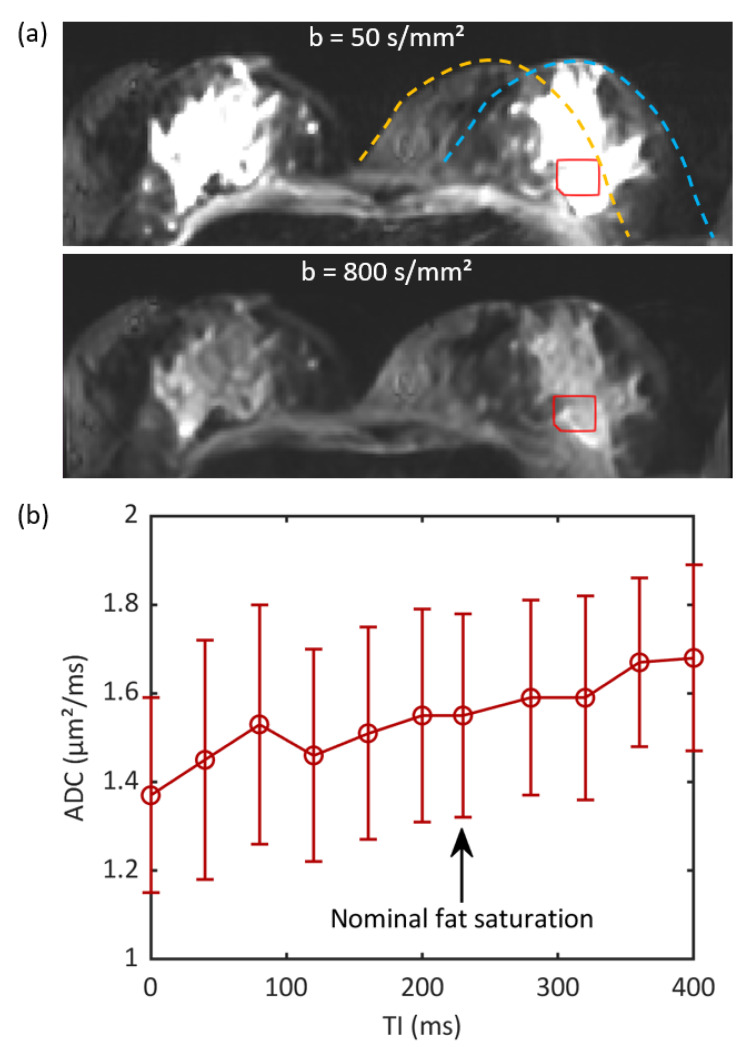
Volunteer experiments. (**a**) Diffusion-weighted images without short-time inversion recovery (STIR) preparation with overlaid segmentations. For demonstration purposes, no spectral fat suppression technique was used. The fat and water signals are represented by the orange and blue dashed lines, respectively. Segmentations encompass regions affected by fat signal contamination (like the red example ROI). (**b**) Mean ± standard deviation of apparent diffusion coefficient (ADC) values across the 10 volunteers. The circular markers represent the mean and the error bars the standard deviation across the 10 volunteers.

For demonstration purposes, the ROIs were manually drawn in fat-contaminated fibroglandular tissue regions (Figure 2a) using the Medical Imaging Interaction Toolkit (version 5.2.1, German Cancer Research Center, Heidelberg, Germany). Using these ROIs, ROI-averaged ADC values were computed using ADC maps derived from the b = 50 s/mm^2^ and b = 750 s/mm^2^ images in MATLAB. Eventually, the mean and standard deviation of ADC values across the volunteers were calculated.

## 3. Results

### 3.1. STIR with Incorrect Inversion Time

Figure 1 summarizes the experiments performed with phantom 1. Figure 1b shows diffusion-weighted images without inversion preparation, with TI = 160 ms and TI = 400 ms. Due to the chemical shift, the oil signal is shifted to the right compared to the syrup signal so that a mixed-signal region arises (red ROI). The blue ROI marks a region with a pure corn syrup solution signal, whereas the yellow ROI marks a region with a pure peanut oil signal. To the left of the yellow ROI, the image appears dark; this is the region where the oil is physically located. It is black because the oil signal is shifted to the right.

Without inversion preparation, in the mixed signal region (red ROI), the oil and syrup signals are in phase, and thus, their signals add up, leading to an overall hyperintense signal compared to the pure syrup region (blue ROI). The ADC map displays a reduced ADC in this mixed signal region. At TI = 160 ms, the oil signal is mostly suppressed, and so the signal in the mixed signal region is almost isointense to the pure syrup region. Similarly, the ADC appears isointense in the mixed signal region and the syrup region. At TI = 400 ms, the oil and syrup signals are out of phase. As a result, the fat contamination in the mixed signal region results in signal hypointensity. Consequently, in the ADC map, an increased ADC is obtained in the mixed signal region.

Figure 1c shows the T_1_ fits (using the b = 50 s/mm^2^ data). The blue and yellow lines represent the T_1_ fit of syrup and oil, respectively. The yellow line assumes its lowest value at approximately TI = 150 ms. The red line is the sum of the other two lines, where the sign of the yellow line was flipped for TI > 150 ms. The reference data points from diffusion-weighted images acquired at TI = 0 ms are shown for completeness but were excluded from the T_1_ fitting procedure, which used data obtained with TI values of ≥40 ms. The T_1_ value obtained was 1.241 s (95% CI: 1.219–1.263) for the syrup and 0.213 s (95% CI: 0.204–0.221) for the oil.

The yellow and blue lines in Figure 1d were set to a constant ADC value obtained in the syrup and the oil compartments, respectively, from the reference scan (i.e., without inversion). The dots correspond to the ADC measurements at different inversion times. The red line represents the two-compartment model. As described in Section 2, this line was not fitted to the ADC data points but generated using Equations (1) and (2) with the parameters previously obtained in the T_1_ fits and the ADC values obtained in the pure signal ROIs (yellow and blue ROIs) in the reference data ($T_{1,syrup}$ = 1.24 s, $T_{1,oil}$ = 0.21 s, ${ADC}_{syrup}$ = 1.09 µm^2^/ms, ${ADC}_{oil}$ = 0.06 µm^2^/ms). The two-compartment model curve closely matches the ADC values in the mixed region that were measured in the red ROI. The two-compartment ADC was smaller than the reference ADC at TI < 150 ms and larger at TI > 150 ms.

Figure 2 presents the volunteer data. Figure 2a shows representative b = 50 s/mm^2^ and b = 750 s/mm^2^ images. The phase encoding direction was left-to-right, resulting in a left shift in the fat signal. This shift is made visible by an orange dashed line that represents the breast contour of the fat image and by a blue dashed line that represents the breast contour of the water image. The manually drawn ROI used for this volunteer is plotted in red; it comprises both fat and water signals.

Figure 2b shows the mean ± standard deviation of measured ADC values across the volunteers. The ADC values generally increased with the TI. The ADC values are generally lower below the nominal fat-saturation inversion time of 230 ms and higher at longer TIs.

### 3.2. STIR with Incorrect Flip Angle

**Figure 3:**
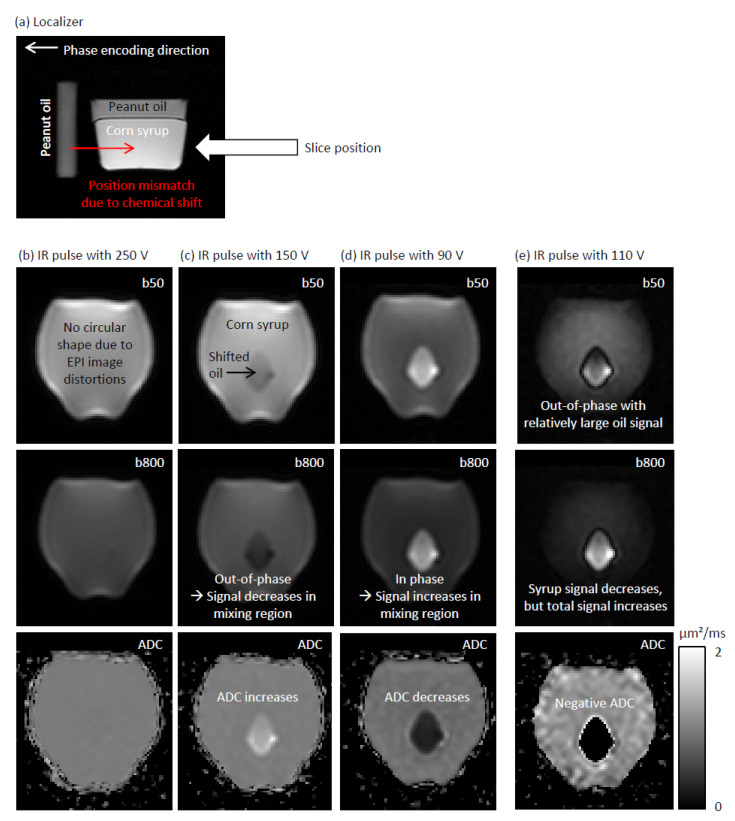
Phantom experiments on an incorrect flip angle in STIR preprations. (**a**) Localizer image. (**b**) Images acquired with inversion pulse voltage of 250 V. (**c**) Inversion pulse voltage = 150 V. (**d**) Inversion pulse voltage = 90 V. (**e**) Inversion pulse voltage = 110 V. b50 = 50 s/mm^2^, b800 = 800 s/mm^2^.

Figure 3 shows the phantom experiments that were performed to illustrate the effect of incorrect flip angles. The localizer image depicted in Figure 3a illustrates the general setup. With the IR pulse voltage of 321 V, the nominal one indicated by the scanner, we observed some small residual oil signal. As Figure 3b shows, a complete suppression of the oil signal was achieved with an IR pulse voltage of 250 V.

In Figure 3c, the oil signal that was shifted into the syrup region becomes visible. In this mixed signal region, an opposed-phase condition arises. The flip angle was so small that the longitudinal magnetization was hardly negative after the IR pulse. At the inversion time, the longitudinal fat magnetization has transitioned from a negative to a positive value, whereas the syrup signal was still negative. This leads an out-of-phase condition and an increase in ADC.

For the even smaller pulse voltage of 90 V (Figure 3d), the flip angle was so small that both the syrup and the oil signal are positive at the inversion time. This in-phase condition leads to an increase in the signal in the mixed signal region and to a reduction in the ADC.

At the pulse voltage of 110 V, the syrup signal is generally smaller than the oil signal and an out-of-phase condition arises. At b = 800 s/mm^2^, the syrup signal decreases considerably so that the total signal increases ($S\left(800\;s/mm^{2}\right)>S\left(0\right)$, compare to Equation (8)):

(10) $$-\left|S_{syrup}\right|\exp\left(-b\cdot D_{tissue}\right)+\left|S_{oil}\right|>-\left|S_{syrup}\right|+\left|S_{oil}\right|$$

for $\left|S_{oil}\right|>\left|S_{syrup}\right|$. Since the signal increases with the diffusion-weighting, the respective ADC is negative.

### 3.3. Spectral Fat Saturation Pulse

**Figure 4:**
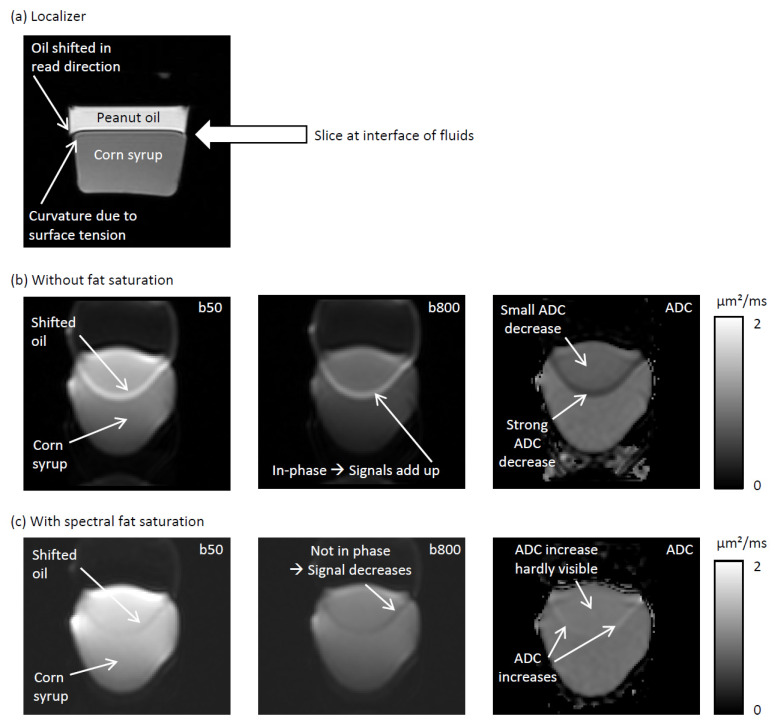
Phantom experiments on the phase of the spectral fat saturation. (**a**) Localizer image. (**b**) Images acquired without any fat saturation. Due to the chemical shift, the oil signal is shifted with respect to the water signal (i.e., with respect to the corn syrup). Owing to the in-phase condition, oil and water signal add up leading to a reduced ADC. (**c**) Images acquired with spectral fat saturation. The oil and the water signal are not in phase resulting in a signal reduction and an increase in ADC.

Figure 4 shows the phantom experiments that were performed to illustrate the effect of the relative phase of water and residual fat signal, which is still present after spectral fat saturation. The general setup is illustrated with the localizer image (Figure 4a). The image slice is placed at the interface region of the two fluids. Due to the surface tension, the surface between the two fluids is curved at the boundaries (“boundary region”), which increases the partial volume of oil in the boundary region. Figure 4b shows images acquired without any fat suppression. The oil and the syrup signal are in phase so that their signals add up. The ADC in the overlap region is reduced. The ADC reduction is larger in the boundary region due to its larger relative fat signal. Figure 4c shows images acquired with spectral fat suppression. The signals of oil and syrup do not add up, instead the overall signal is reduced in the respective regions, which leads to an ADC increase.

## 4. Discussion

Contamination of the water signal by residual fat signal is commonly believed to result in underestimation of the ADC [21,22,23,24,25]. However, the demonstrative phantom and volunteer experiments in our study showed that the residual fat signal can lead to an overestimation of the ADC under out-of-phase conditions, where the fat and water signals have opposite polarities. The underlying reason is that the spurious fat signal affects the high-b-value signal more than the low-b-value signal (in relative terms). If the low-b-value signal is assumed to be approximately unaffected, then an increased high-b-value signal that arises in an in-phase condition leads to an underestimation of the ADC. Conversely, a decreased high-b-value signal in an out-of-phase condition leads to an overestimation of the ADC.

Several studies have already compared different fat suppression techniques and their influence on the measured ADC. For example, Stadlbauer et al. investigated the differences between STIR and spectral presaturation with inversion recovery (SPIR) fat suppression methods [38]. They observed that ADC values obtained with STIR were consistently higher than those obtained with SPIR. They attributed this discrepancy primarily to differences in T_1_-weighting between the intracellular and extracellular compartments, which is a plausible explanation. However, our findings suggest that the observed difference may also be partially attributed to the overestimation of the ADC in STIR-DWI due to residual spurious fat signal. This mechanism offers an additional explanation for the elevated ADC values seen with STIR-based fat suppression.

In SPIR fat suppression—and its adiabatic variant, spectral attenuated inversion recovery (SPAIR)—the fat magnetization is selectively inverted, similar to STIR, but the water magnetization remains unaffected. Thus, the water signal remains positive with SPIR and SPAIR but is negative with STIR at the same inversion time. Consequently, for a given flip angle and inversion time, an in-phase signal condition in STIR can become out-of-phase in SPIR or SPAIR and vice versa. This phase reversal implies that an overestimated ADC in STIR-DWI (due to fat–water cancellation effects) may correspond to an underestimated ADC in SPIR/SPAIR-DWI and vice versa. Therefore, the differences in ADC values between STIR and SPIR reported by Stadlbauer et al. could be at least partially explained by a combination of STIR-ADC overestimation and SPIR-ADC underestimation, both due to residual fat signal interactions.

Several studies have reported findings consistent with those of Stadlbauer et al. [38]. Wenkel et al. [20] compared spectral and STIR fat suppression in breast DWI, finding higher ADC values with STIR, particularly in benign lesions. Similarly, Baron et al. evaluated four fat suppression techniques in breast DWI—STIR, SPAIR, spectral, and water excitation—and also reported the highest ADC values with STIR [39]. Mürtz et al. [22] observed higher ADC values with STIR than SPIR in both benign and malignant breast lesions, while Nogueira et al. [40] reported higher ADC values with STIR than with SPAIR across benign and malignant lesions as well as normal breast tissue. In contrast, Kazama et al. [34] found slightly lower ADC values when using STIR-DWI compared to a spectrally selective fat suppression technique.

Most of the mentioned studies reported higher ADC values with STIR fat suppression than with other fat suppression techniques. This observation can be explained by our analysis, which shows that STIR fat suppression can lead to overestimation of ADCs.

In our demonstration experiments, the chosen range of TI values was exaggerated to clearly demonstrate the effects. However, the task of choosing the correct TI is not a trivial one in practice. One problem is that the reported fat T_1_ times vary among publications. For example, the review article by Bojorquez et al. summarizes reported values ranging from 366 ms to 450 ms at 3 T [41]. Moreover, different investigators have used different inversion times for STIR fat suppressions (e.g., at 1.5 T: 150 ms [20], 180 ms [22,38], 185 ms [39]). Even among the vendor-suggested sequences of a single scanner, the inversion times for STIR fat suppressions may vary. For example, our 3 T Magnetom Cima.X scanner (Siemens Healthineers, Erlangen, Germany, syngo version XA61) provides vendor-suggested sequences with:

- TI = 220 ms (“t2_tse_stir_tra” in the breast library for medium-channel coils).
- TI = 230 ms (“t2_tse_stir_tra” in the breast library for high-channel coils).
- TI = 240 ms (“ep2d_diff_stir_b50-800_tra” in the whole body diffusion folder).

Different repetition times of the different sequences may not explain the different inversion times (TIs) because TR was larger than 3 s in all cases (i.e., in the cited studies and the mentioned Cima.X sequences) and therewith had little influence on the inversion time setting. This demonstrates that it is unlikely that the perfect inversion time is hit in all investigations and some influence of TI on the $D_{partial\;volume}$ may be present in practice.

Especially at higher field strengths, the $B_{1}^{+}$ field is not perfectly homogeneous and generates spatially varying flip angles within the imaging volume [42,43]. The factor $c_{fat}$ has a cosine-dependency on the flip angle error Δα (see Equation (6)). Since $\cos\left(\Delta \alpha \right)\approx 1-\Delta \alpha^{2}$ depends only to second order on Δα, small flip angle errors only have a small effect of the measured $D_{partial\;volume}$. This is presumably also the reason why we had to choose rather large voltage changes in the respective demonstration experiments (see Figure 3) to make the in-phase and out-of-phase effects visible. However, if incorrect $B_{1}^{+}$ field amplitudes are an issue, one option is to use an inversion time that is shorter than the nominally correct value to achieve better volume-averaged fat suppression. In that case, a single dataset might suffer from both ADC underestimation and overestimation with STIR fat suppression.

We could also demonstrate that out-of-phase conditions may arise with spectral fat suppressions (Figure 4). The occurrence of these out-of-phase conditions in practice will depend on the effective pulse phase difference in a particular sequence at a particular field strength—and on the actually applied flip angle. In particular, flip angles surpassing 90° due to an imperfect calibration will generate a phase flip. Potentially, these conditions have not been so prevalent in previous studies, which could explain the above discussed generally lower ADC values found with spectral fat suppressions compared to STIR compressions.

As Le Bihan et al. noted, “fat contamination may lead to false-positive cases” [44], as the ADC value of a benign lesion can be artificially reduced by the fat signal, potentially leading to its misclassification as a low-ADC malignant lesion. In contrast, the potential overestimation of the ADC that we describe here can have the opposite effect: it may result in false-negative cases, where the ADC value of a malignant lesion is elevated to the point that it is incorrectly classified as benign.

Le Bihan et al. [44] comprehensively compared STIR and SPAIR fat suppression techniques, addressing a broad range of topics beyond the scope of our study, including differences in signal-to-noise ratio and specific absorption rate. Notably, they also reported instances of ADC overestimation with STIR fat suppression, which they primarily attributed to T_1_ heterogeneity within lesions. However, their finding essentially also explains the fat-induced overestimation of the ADC with STIR fat suppression that we considered here (in the analysis of the effect of incorrect inversion times).

In addition to evaluating ADC maps and values, a common strategy in DWI is to inspect diffusion-weighted images acquired with b-values >1000 s/mm^2^ to identify malignant lesions. At these high b-values, malignant lesions typically appear hyperintense due to their low ADC values. This approach is most widely used in prostate DWI [45,46,47], but it is also employed in other fields, such as breast DWI [48,49]. At these high b-values, spectral fat suppression techniques may fail since they do not suppress the signal from olefinic protons. In this context, STIR fat suppression becomes especially important for achieving reliable fat signal suppression. However, with STIR fat suppression, an out-of-phase condition (as described by Equation (8)) can result in a reduction in lesion intensity due to fat–water signal cancellation (see Figure 1, TI = 400 ms, the hypointense region at b = 800 s/mm^2^). This effect may compromise lesion conspicuity and potentially result in false-negative findings, particularly in clinical scenarios where lesion visibility is crucial.

The out-of-phase condition that leads to the overestimation of the ADC can also occur under different circumstances. Beyond the realm of fat suppressions, out-of-phase conditions may also arise in inversion recovery experiments when a voxel contains compartments with different T_1_ times. For example, in prostate MRI, an out-of-phase relationship between prostate fluid and tissue has been reported [50]. In this case, however, the effect is reversed: the tissue ADC is underestimated rather than overestimated because, unlike the ADC of fat, the ADC of prostate fluid is higher than the ADC of the surrounding tissue. Thus, partial signal cancellation under out-of-phase conditions leads to an apparent reduction in the tissue’s measured ADC in a partial volume setting with high-ADC fluid contamination.

Our study had some limitations. Firstly, we only measured phantoms and volunteers but did not perform patient examinations. Thus, we could not directly demonstrate the potential masking effect that an out-of-phase condition may have on lesion visibility in very high b-value images. Among the high-b-value (1500 s/mm^2^) images acquired in healthy volunteers, we unfortunately could not identify a suitable example and thus did not further evaluate the b = 1500 s/mm^2^ data. It is not entirely surprising that we did not find a suitable example image since one of the primary goals of using such high b-values is to suppress the signal from fibroglandular tissue [48,49]. Secondly, we did not systematically assess the prevalence or spatial distribution of out-of-phase conditions in the volunteer datasets. A potential approach for future studies would be to perform B_1_^+^ mapping [43,51] and examine correlations between local flip angles and measured diffusion coefficients, thereby quantifying the impact of B_1_^+^ inhomogeneities on ADC estimation. Thirdly, we did not evaluate other techniques, such as SPAIR and SPIR, despite the fact that out-of-phase effects can also occur with these methods. We limited our analysis to STIR because the TI could be adjusted within the sequence we used, whereas it could not be adjusted for SPAIR, and SPIR was unavailable in the sequence we used. Fourthly, in our discussion, we neglected that diffusion-weighted images are prone to quasi-random phase instabilities that arise from pulsatile motion of the tissue [52]. If fat signal is shifted into such an instable phase region, the simple “plus/minus” picture painted in the theory section does not adequately capture reality anymore. Instead, the problem becomes more complicated and one would have to consider the exact phase relation between fat and tissue signals. Such a situation will most likely occur when subcutaneous fat shifts into adjacent organs like the brain or the liver. Such a quasi-random phase situation may also affect our breast data although presumably to a lesser extent. After all, the quasi-random phases are known to vary smoothly across the image [52]. Fifth, our pulse voltage experiments were only qualitative. We had difficulties in linking the applied voltage to the flip angle. Potentially, this issue arose because we hijacked the individual pulse voltage setting in fashion not anticipated by the manufacturer, who uses RF pulses that are numerically optimized for certain flip angle settings. For example, we would have expected the setting shown in Figure 3e to appear at a much smaller pulse voltage. Lastly, we focused on the apparent diffusion coefficient in our analysis and neglected more sophisticated metrics such as those of intravoxel incoherent motion imaging [53], diffusion kurtosis and diffusion tensor metrics [54]. An analysis of such metrics would be an interesting topic of future studies.

## 5. Conclusions

In conclusion, we demonstrated that out-of-phase conditions can lead to the overestimation of tissue ADC in the presence of a residual fat signal. This effect may result in false-negative lesion classifications, particularly when malignant lesions appear to have elevated ADC values. Out-of-phase conditions arising from incomplete fat suppression may also reduce lesion conspicuity in high b-value images, potentially masking clinically relevant findings.

## Figures and Tables

**Table 1:** Acquisition parameters.

Parameter	Volunteer Acquisition (TI)	Phantom (TI)	Phantom (Flip Angle)	Phantom (Spectral Fat Suppression)
No. Slices	15	1	1	1
Slice thickness (mm)	4.0	5.0	5.0	5.0
Repetition time (TR; ms)	7230	8400	2000	2000
Echo time (TE; ms)	65.00	101.00	101.00	101.00
Inversion time (TI; ms)	40, 80, 120, 160, 200, 230, 280, 320, 360, 400; and one dataset without inversion preparation	0, 40, 80, …, 400; and one dataset without inversion preparation	180	No inversion
Spectral fat suppression	Yes (and gradient reversal)	Yes (and gradient reversal)	No	Once with and once without
b-values ($s/{mm}^{2}$) [repetitions]	50 [3], 750 [8], 1500 [15]	50 [1], 800 [2]	50 [1], 800 [2]	50 [1], 800 [2]
Diffusion mode	3D Diagonal	4-Scan Trace	4-Scan Trace	4-Scan Trace
Voxel size (${mm}^{3}$)	3 × 3 × 4.0	1.8 × 1.8 × 5.0	2.2 × 2.2 × 5.0	2.2 × 2.2 × 10.0
Acquisition bandwidth (Hz/Px)	2298	1624	1686	1624
Echo spacing (ms)	0.54	0.77	0.68	0.68
Orientation	Transversal	Transversal	Coronal	Coronal
Field of view (FOV; mm)	390	200	250	200
Phase encoding direction	L >> R (right to left)	L >> R (right to left)	H >> F (head to feet)	H >> F (head to feet)
FOV phase	81.3%	63.2%	42.1%	42.1%
Base resolution	128	114	114	114
Phase resolution	100%	100%	100%	100%
Parallel imaging	GRAPPA ×2	No parallel imaging	No parallel imaging	No parallel imaging
Deep Resolve	On	Off	Off	Off
Deep Resolve Boost	On	Off	Off	Off
Scan time	3 min 30 s (3 b-values)	1 min 59 s (2 b-values, per TI)	32 s (2 b-values, per voltage)	2 × 32 s (2 b-values)
Gradient Mode	Performance	Fast	Fast	Fast

GRAPPA: generalized autocalibrating partially parallel acquisitions, 3D diagonal = gradient direction (1, 1, 1) in scanner coordinate system. All scans were performed with a manufacturer-provided single refocused echo planar imaging (EPI) DWI sequence, the “prescan normalize” surface coil flare correction, and the “dynamic field correction” method that minimizes eddy current induced EPI image distortion. The approach to switch of the spectral fat saturation was to set the respective pulse voltage to zero.

## Data Availability

The data generated or analyzed during this study are available from the corresponding author upon reasonable request.

## Abbreviations

The following abbreviations are used in this manuscript:

ADC	Apparent diffusion coefficient
CI	Confidence interval
DWI	Diffusion-weighted imaging
GRAPPA	Generalized autocalibrating partially parallel acquisitions.
MRI	Magnetic resonance imaging
ROI	Region of interest
TI	Inversion time
STIR	Short-time inversion recovery
